# Dual Regulation of the Rice Yield Traits and Stress Resilience by *OsHSP20*: Insights from Integrative Biochemical and Transcriptomic Analyses

**DOI:** 10.3390/ijms27188341

**Published:** 2026-09-19

**Authors:** Yan Liao, Muneeba Saleem, Nan Zhang, Qingping Pang, Juan Du, Qianhui Wang, Siyao Li, Baishi Chen, Qiong Hu, Yuanyuan Nie, Lin Zhang, Hanhua Tong, Zhen Zhang, Haohua He, Songping Hu

**Affiliations:** 1 Research Center for Plant Functional Genes and Tissue Culture Technology, College of Bioscience and Bioengineering, Jiangxi Agricultural University, Nanchang 330045, China; liaoyan200105@163.com (Y.L.); mooneeba78@gmail.com (M.S.); z18794800748@126.com (N.Z.); wanlimepqpy@163.com (Q.P.); lsdujuan@163.com (J.D.); lsy15144728392@163.com (S.L.); 18867425265@163.com (Q.H.); zhanglin06@163.com (L.Z.);; 2 Super Rice Research and Development Center, Jiangxi Academy of Agricultural Sciences, Nanchang 330200, China; happynyy@163.com; 3 State Key Laboratory of Rice Biology and Breeding, China National Rice Research Institute, Hangzhou 311410, China; htonghz@126.com; 4 Agriculture College, Jiangxi Agricultural University, Nanchang 330045, China; hhhua64@163.com

**Keywords:** *Oryza sativa*, *OsHSP20*, gene function, seed setting rate, stress response, high photosynthetic rate

## Abstract

Rice (*Oryza sativa* L.) is a staple food crop globally, and identifying genes governing grain yield is critical for food security. Although heat shock proteins (HSPs) are known for their roles in stress tolerance, the molecular mechanisms by which they regulate yield formation remain unclear. In this study, we generated knockout and overexpression lines for *OsHSP20* which is encoding a member of the Hsp20/alpha crystallin family, *LOC_Os10g30162.1* (It is also one of the four candidate genes discovered during our fine mapping of major QTLs for photosynthetic rate in rice.), and performed integrated analyses combining field phenotyping, multi-stress assays, and transcriptomics. Phenotypic analyses revealed that *OsHSP20* deficiency resulted in compromised plant architecture, leaf morphology, tillering, and panicle development, leading to a significant reduction in grain setting rate. Conversely, *OsHSP20* overexpression enhanced drought tolerance. Mechanistically, transcriptomic and functional analyses demonstrated that *OsHSP20* maintains protein homeostasis under drought stress via its chaperone activity; this function orchestrates a coordinated regulatory network involving lipid barrier formation, antioxidant defense, and carbon allocation. Our findings establish *OsHSP20* as a positive regulator of both yield and drought resilience, improving crop adaptability by balancing growth and stress responses. In addition, our previous research has shown that *OsHSP20* is actually one of the important components of the main QTL for rice photosynthetic rate. Therefore, this study can provide new genetic resources and a theoretical basis for cultivating rice varieties with high yield, stress resistant, and a high photosynthetic rate.

## 1. Introduction

With the ongoing deterioration of the global climate and environment, coupled with anthropogenic disturbances, arable land area is progressively declining, thereby constraining food production and the sustainable development of human society. As one of the three most important staple crops worldwide, enhancing rice yield remains a paramount strategy for safeguarding food security and meeting the escalating demands of the global population [1].

The identification and utilization of novel gene resources intimately linked to yield formation represent a critical avenue for achieving continued increases in rice productivity [2]. Rice yield is collectively determined by four components: effective panicle number, filled grains per panicle, 1000-grain weight, and grain setting rate. Optimal yield is achieved only when these components reach a specific equilibrium [3]. Among these, 1000-grain weight is relatively stable, primarily attributable to its high heritability. In contrast, the remaining three components exhibit substantial variability influenced by both genetic and environmental factors. While effective panicle number and filled grains per panicle demonstrate compensatory effects on yield, grain setting rate exhibits relatively low heritability and is predominantly modulated by environmental factors such as soil conditions and light intensity. Its stability and magnitude correlate positively and significantly with yield stability [4]. Defined as the percentage of plump grains relative to total spikelets, grain setting rate constitutes a pivotal determinant of rice yield, second only to panicle number per plant. Enhancing grain setting rate directly augments the number of filled grains per panicle, thereby increasing overall yield. Currently, hybrid rice frequently exhibits a marked decline in grain setting rate compared to its parental lines; thus, a low grain setting rate has emerged as a limiting factor or bottleneck impeding the further exploitation of heterosis for yield improvement. Consequently, elucidating the molecular genetic mechanisms governing grain setting and identifying novel favorable alleles controlling this trait hold profound significance for boosting rice productivity.

Heat shock proteins (HSPs) constitute a class of proteins abundantly expressed in response to environmental stressors. They play essential roles in maintaining protein homeostasis, facilitating the refolding of denatured proteins, and enhancing stress tolerance [5]. Existing evidence indicates that HSPs function as both essential “housekeeping” proteins and stress-defense proteins, exhibiting diverse biological functions including molecular chaperoning, stress defense, regulation of plant growth and development, modulation of signal transduction pathways, and cross-protection under various adverse conditions [6].

In our previous study we discovered that the rice *OsHSP20* gene is one of the four candidate genes for the major QTL-qPR10 affecting rice photosynthetic rate. These four candidate genes are as follows: *LOC_Os10g30156.1* encoding starch synthase II or chloroplast precursor, *LOC_Os10g30162.1* encoding heat shock protein Hsp20 protein binding domain, *LOC_Os10g30560.1* encoding UDP-glucuronosyl/UDP-glucosyltransferase protein family, and *LOC_Os10g31320.1* with unknown function. Then we conducted a detailed study on the seed setting rate and stress resistance physiological functions of the *OsHSP20* gene (*LOC_Os10g30162.1*) under adverse conditions such as drought and heat stress. We characterized a T-DNA insertion mutant, *oshsp20*, which exhibits reduced expression of the *OsHSP20* gene. Field observations revealed that *oshsp20* mutants displayed a significant reduction in grain setting rate compared to the wild-type cultivar. The latest progress in research on the *OsHSP20* gene was conducted by Bian Jianmin [7]. They believe that the *OsHSP20* gene is not only related to drought resistance but also to heat resistance. However, they have not conducted research on the relationship between this gene and seed setting rate. So our aims are to elucidate the expression profiles of *OsHSP20* across various rice tissues using molecular and cytological analyses, thereby establishing a foundation for deciphering its biological function and providing theoretical guidance for the stable, high-yield, high-resistance breeding of rice.

## 2. Results

### 2.1. OsHSP20 Exhibits Pleiotropic Effects on Plant Architecture

**Figure 1:**
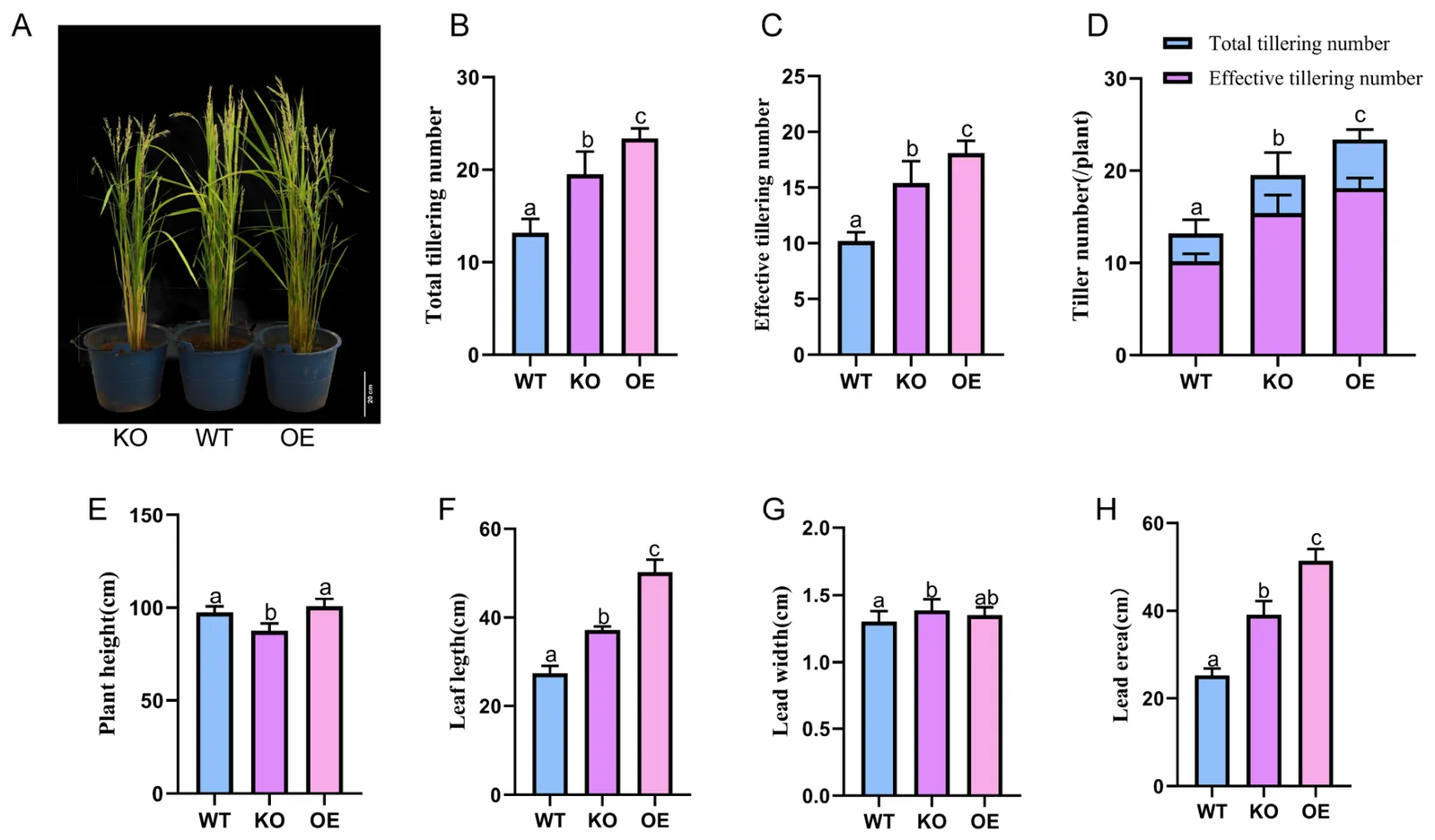
Phenotypic investigation of *OsHSP20* transgenic lines. (**A**) Transgenic line plants at the grain-filling stage, scale bar = 20 cm; (**B**) total tiller number; (**C**) effective tiller number; (**D**) tiller number per plant; (**E**) plant height; (**F**) leaf length; (**G**) leaf width; (**H**) leaf area. (n = 10).

At the grain-filling stage, *OsHSP20*-KO lines exhibited increased tiller numbers but reduced plant height compared to the wild type (WT), while *OsHSP20*-OE lines showed further enhanced tillering and leaf expansion (Figure 1). However, the increase in ineffective tillers resulted in a stable panicle formation rate, indicating that *OsHSP20* primarily regulates tiller quantity rather than quality. Unlike typical loss-of-function phenotypes, the heightened tillering in KO lines suggests involvement in a complex compensatory network (Figure 1B–D). Additionally, *OsHSP20* is essential for stem elongation, as KO lines displayed pronounced dwarfism, whereas OE lines showed no additional height gain, implying a dosage saturation effect (Figure 1E). Leaf morphology analysis revealed that *OsHSP20* promotes leaf expansion; OE lines exhibited significantly larger leaf areas, likely due to its role as a molecular chaperone in cell wall biosynthesis or division [8] (Figure 1F–H). The differential regulation of leaf length and width underscores the gene’s spatial specificity in growth control.

### 2.2. OsHSP20 Modulates Panicle Architecture and Yield Components

**Figure 2:**
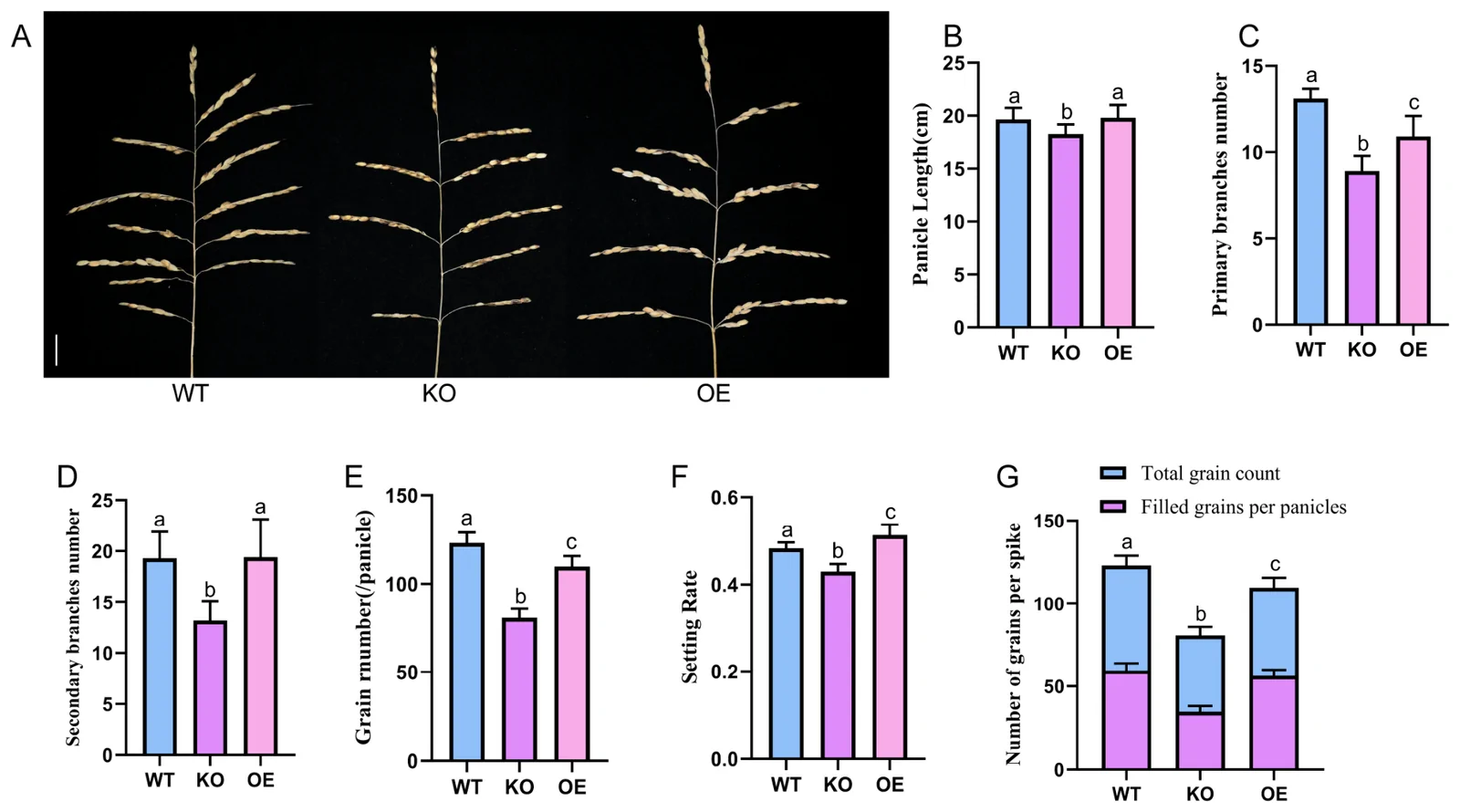
Panicle traits of *OsHSP20* transgenic lines. (**A**) Main panicle, scale bar = 2 cm; (**B**) main panicle length; (**C**) number of primary branches; (**D**) number of secondary branches; (**E**) number of grains per panicle; (**F**) seed setting rate; (**G**) total grain number and filled grain number. (n = 10).

Knockout of *OsHSP20* caused a comprehensive deterioration of panicle architecture, including shortened panicles and reduced primary and secondary branches, leading to a drastic decline in total and filled grains per panicle (Figure 2). While overexpression (*OsHSP20*-OE) partially restored primary branch number, it failed to fully rescue total grain number (Figure 2B,C). Intriguingly, OE lines exhibited a significantly higher grain setting rate than the wild type (WT), despite bearing fewer total spikelets. This trade-off—enhanced grain filling efficiency at the cost of reduced spikelet number—aligns with the altered panicle architecture and increased ineffective tillers observed previously. These results demonstrate that *OsHSP20* exerts pleiotropic effects on yield components by balancing reproductive merit with resource allocation.

### 2.3. OsHSP20 Affects Grain Morphogenesis and Filling Capacity

**Figure 3:**
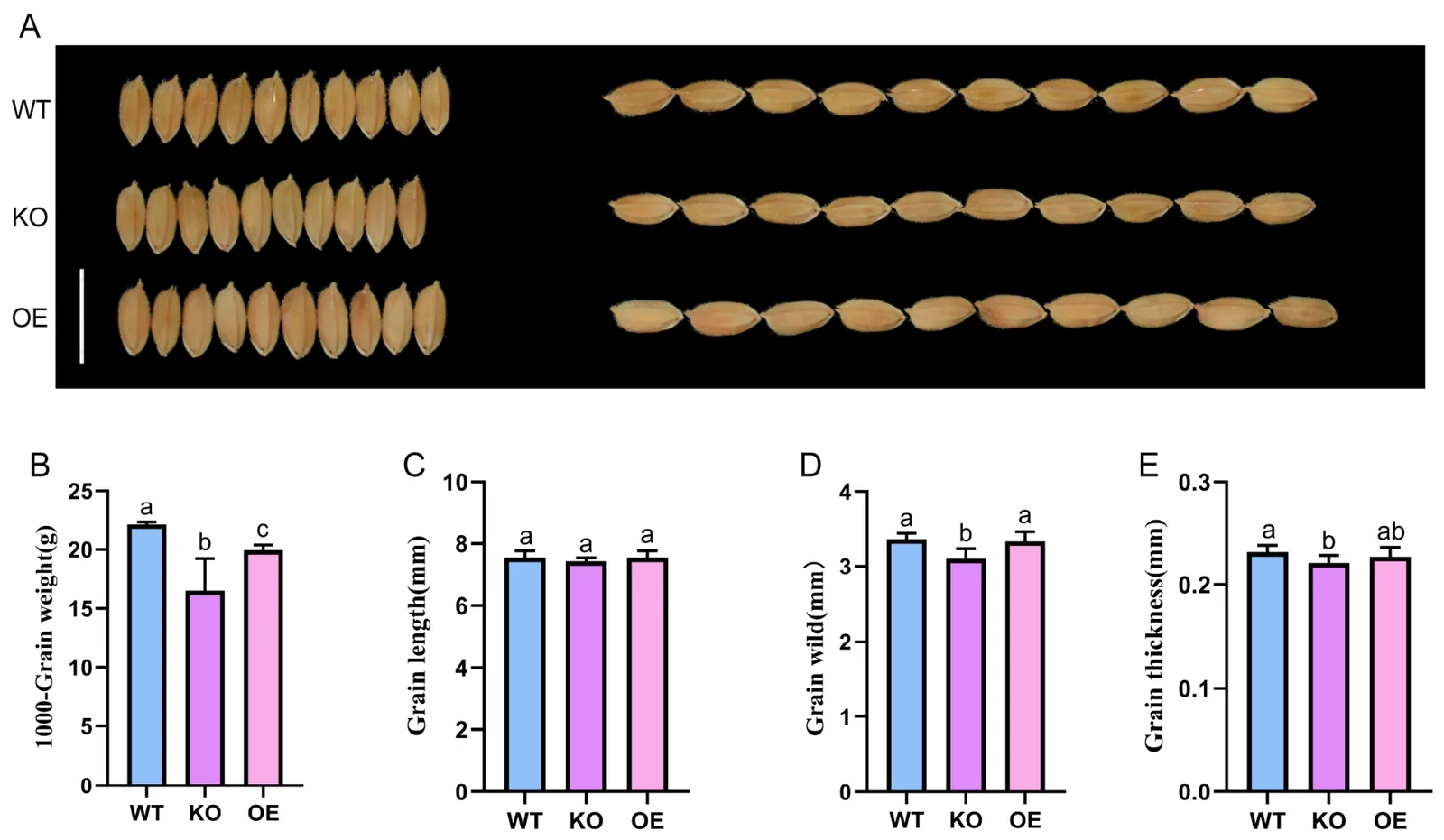
Phenotypic investigation of *OsHSP20* transgenic lines. (**A**) Grain, scale bar = 1 cm; (**B**) thousand-grain weight; (**C**) grain length; (**D**) grain width; (**E**) grain thickness. (n = 10).

*OsHSP20* deficiency resulted in a specific reduction in grain width, thickness, and 1000-grain weight, while grain length remained unchanged (Figure 3). Overexpression partially rescued the 1000-grain weight but did not fully restore it to WT levels. This selective impairment of grain girth and filling capacity aligns with the panicle defects, confirming that *OsHSP20* is crucial for establishing grain morphology and sink strength during seed development.

### 2.4. OsHSP20 Modulates Rice Germination Under Drought Stress

**Figure 4:**
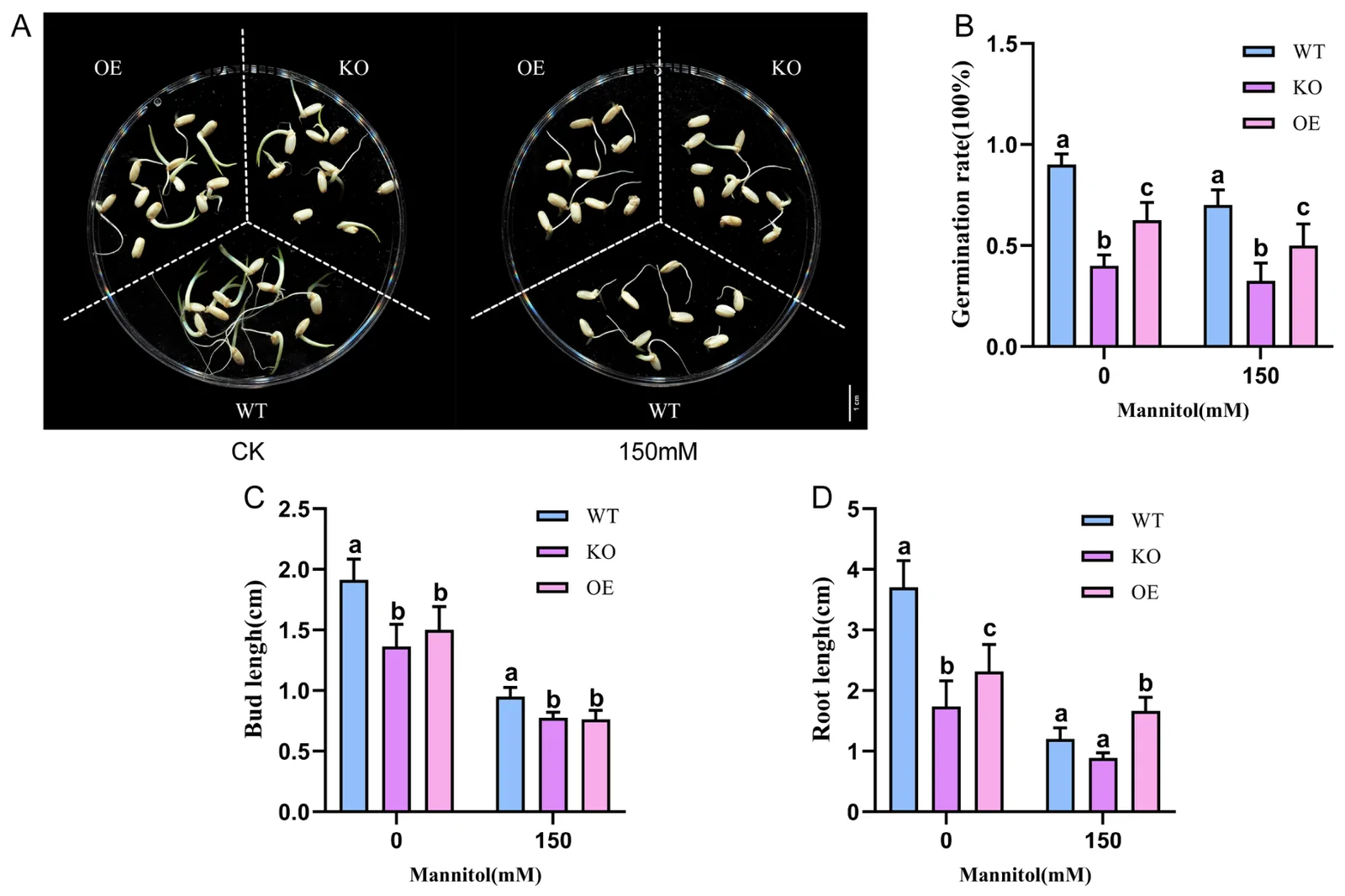
Drought germination assay of *OsHSP20* transgenic lines. (**A**) Growth performance of *OsHSP20* transgenic lines after 3 days of germination, scale bar = 1 cm; (**B**) seed germination rate; (**C**) shoot length; (**D**) root length. (n = 10).

Under normal conditions, knockout of *OsHSP20* severely impaired seed germination, while overexpression (*OsHSP20*-OE) partially rescued this defect (Figure 4). Under 150 mM mannitol-induced drought stress, *OsHSP20*-KO lines exhibited the lowest germination rate (31%) and shortest roots. Conversely, *OsHSP20*-OE lines maintained significantly longer roots than the wild type (WT), demonstrating a mitigation of drought-induced inhibition. These results indicate that *OsHSP20* is crucial for maintaining root system architecture under drought stress during early seedling establishment.

### 2.5. OsHSP20 Modulates Post-Germination Growth in Rice Under Drought Stress

**Figure 5:**
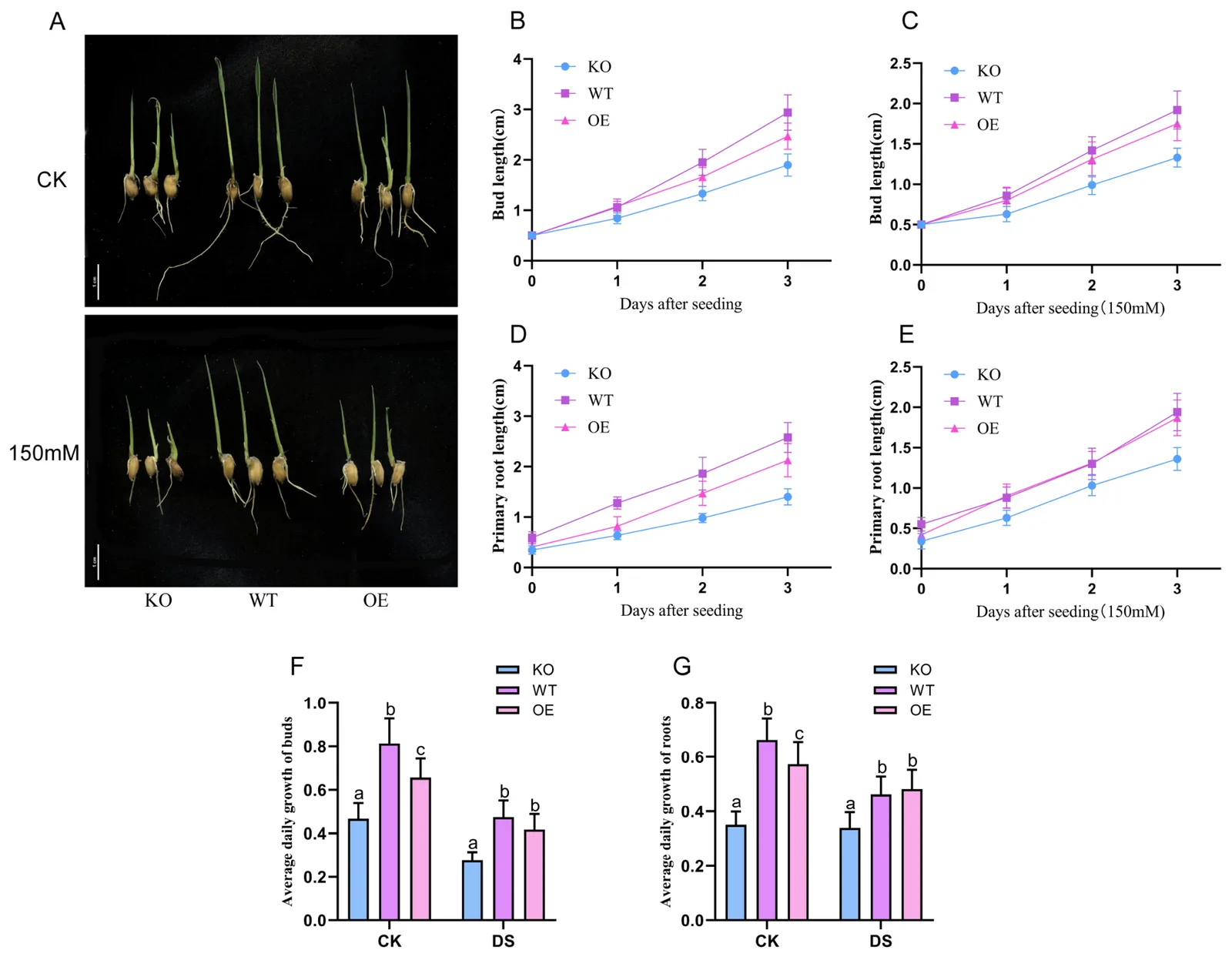
Post-germination drought stress experiment of *OsHSP20* transgenic lines. (**A**) Growth performance of transgenic lines after 3 days of hydroponic culture, scale bar = 1 cm; (**B**) shoot length statistics in the control group; (**C**) shoot length statistics in the treatment group; (**D**) root length statistics in the control group; (**E**) root length statistics in the treatment group; (**F**) average daily increase in shoot length; (**G**) average daily increase in root length. (n = 10).

Hydroponic assays simulating drought stress (150 mM mannitol) revealed that *OsHSP20* is critical for post-germinative growth (Figure 5). Under normal conditions, knockout (*OsHSP20*-KO) lines exhibited significantly impaired shoot and root elongation rates compared to the wild type (WT), while overexpression (*OsHSP20*-OE) lines showed partial restoration. Under drought stress, *OsHSP20*-KO lines displayed the most severe growth inhibition. Conversely, *OsHSP20*-OE lines effectively mitigated drought-induced repression; notably, the root growth rate in *OsHSP20*-OE lines (0.48 cm/day) was comparable to or slightly exceeded that of the WT (0.46 cm/day), whereas the *OsHSP20*-KO lines lagged significantly (0.34 cm/day).

These results demonstrate that *OsHSP20* positively regulates seedling establishment. Its overexpression enhances drought resilience by maintaining root system architecture and growth rate, highlighting its protective role in early development under drought stress.

### 2.6. OsHSP20 Orchestrates Biomass Allocation and Water Homeostasis Under Drought Stress

**Figure 6:**
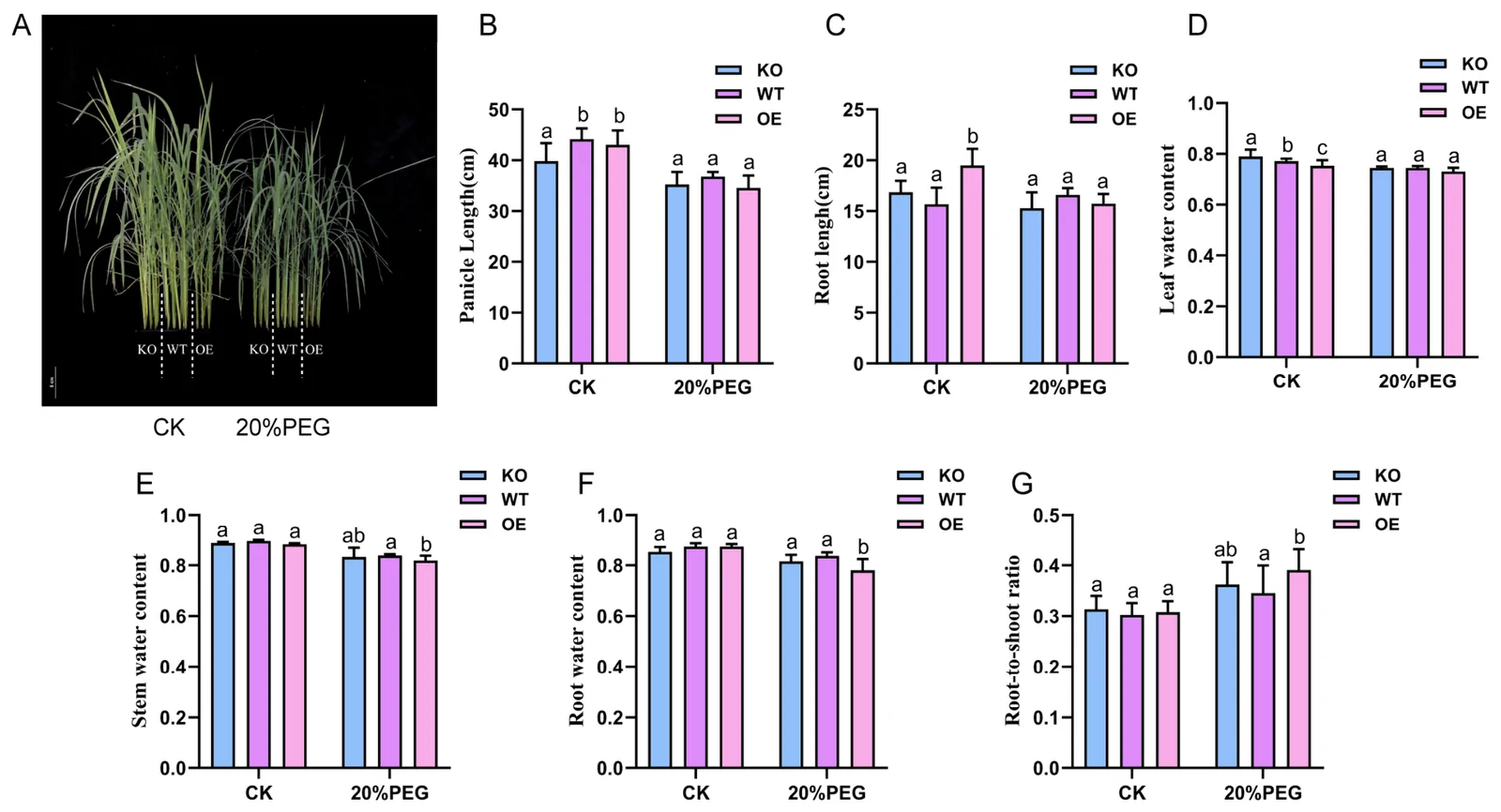
Seedling-stage drought treatment of *OsHSP20* transgenic lines. (**A**) Growth status of the control and treatment groups after 5 days, scale bar = 5 cm; (**B**) plant height; (**C**) root length; (**D**) leaf water content; (**E**) stem water content; (**F**) root water content; (**G**) root-to-shoot ratio. (n = 10).

Two-week-old seedlings were subjected to 20% PEG-induced drought stress for five days. Under stress, *OsHSP20*-KO lines exhibited stunted growth, while *OsHSP20*-OE lines displayed a significant reduction in plant height compared to the wild type (WT) (Figure 6). This growth suppression correlated with a notable increase in the root-shoot ratio specifically in the OE lines, suggesting a strategic shift in biomass allocation to prioritize root development and minimize transpiration. Furthermore, tissue water content analysis revealed that OE lines maintained lower stem and root moisture levels under stress compared to WT and KO lines. These results indicate that *OsHSP20* modulates drought adaptation by fine-tuning growth patterns and tissue-specific water retention, thereby enhancing resource-use efficiency during seedling establishment.

### 2.7. OsHSP20 Maintains Photosynthetic Efficiency and Redox Homeostasis Under Drought Stress

**Figure 7:**
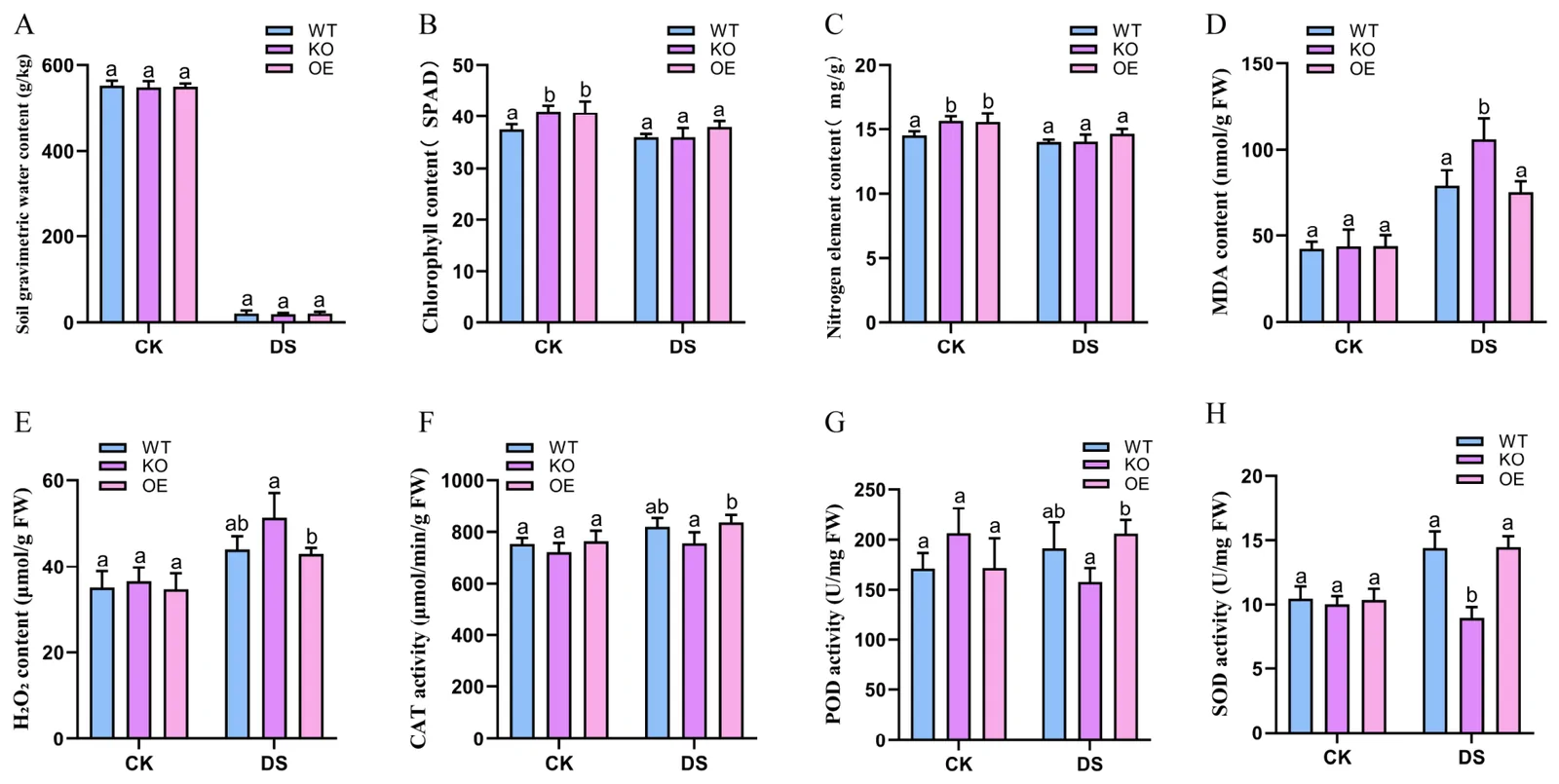
Physiological and biochemical indicators of *OsHSP20* transgenic lines under soil drought treatment. (**A**) Soil water content; (**B**) chlorophyll content; (**C**) nitrogen content; (**D**) MDA content; (**E**) H_2_O_2_ content; (**F**) CAT activity; (**G**) POD activity; (**H**) SOD activity. (n = 3).

Soil drought assays at the tillering stage revealed that *OsHSP20* is critical for stress resilience (Figure 7). Under equivalent soil water deficits, *OsHSP20*-KO lines exhibited severe membrane damage, as evidenced by elevated malondialdehyde (MDA) and hydrogen peroxide (H_2_O_2_) accumulation. This was attributed to a dysfunctional antioxidant system, characterized by suppressed superoxide dismutase (SOD) and peroxidase (POD) activities. Conversely, *OsHSP20*-OE lines sustained higher SOD and POD activities, effectively scavenging reactive oxygen species (ROS) and minimizing oxidative injury. Consequently, OE lines maintained superior chlorophyll and nitrogen content compared to the wild type (WT) and KO lines. These findings demonstrate that *OsHSP20* enhances drought tolerance by orchestrating antioxidant defense and preserving photosynthetic capacity. This also explains, at the molecular level, why the heat shock protein gene *OsHSP20* can be one of the four candidate genes for the major QTL of photosynthetic rate in our previous study.

### 2.8. OsHSP20 Enhances Thermotolerance by Modulating Antioxidant Defense and Photosynthetic Stability

**Figure 8:**
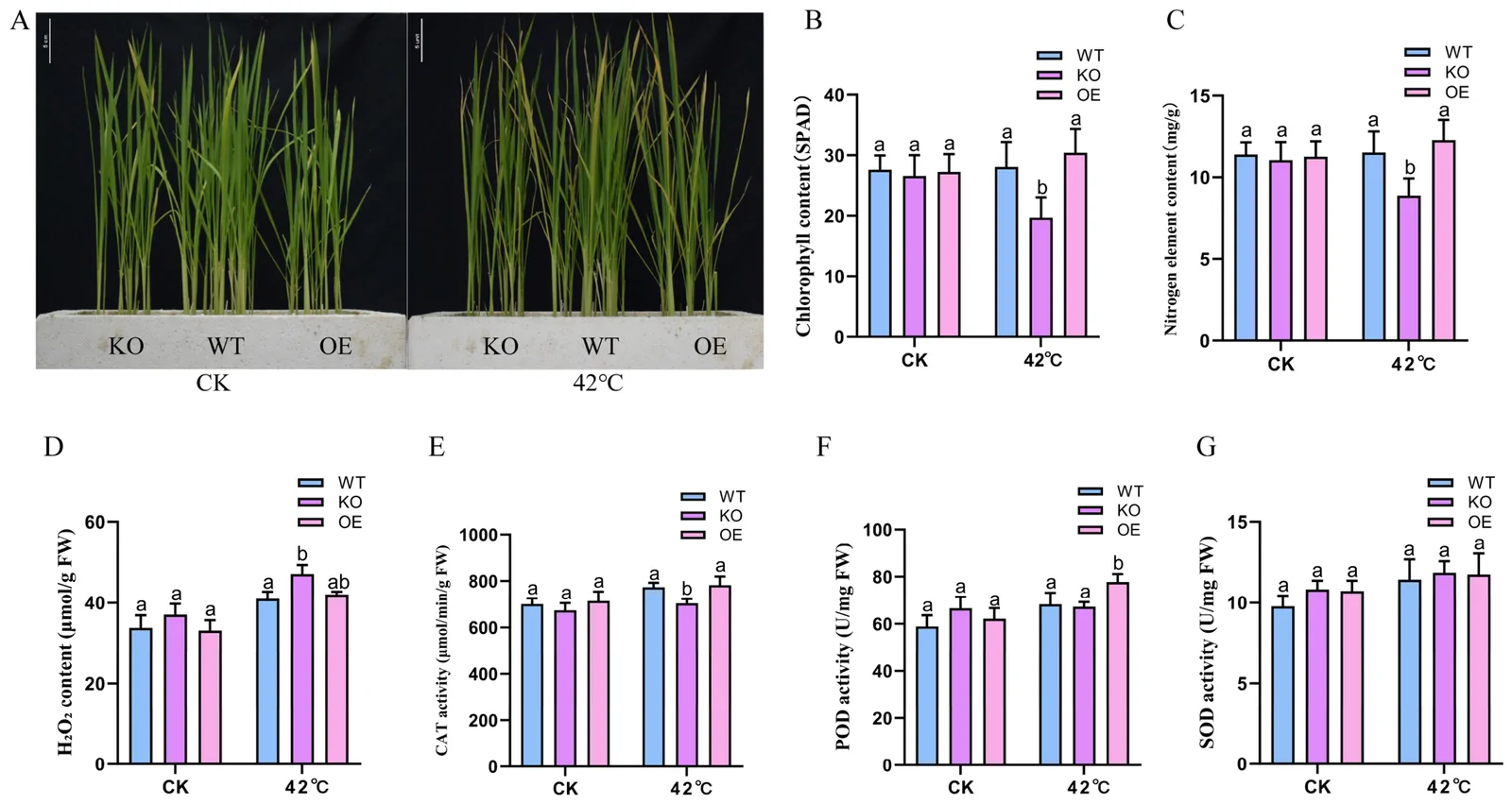
Physiological and biochemical Indicators of *OsHSP20* transgenic lines under high temperature treatment. (**A**) Growth status of control and treatment groups after 3 days, scale bar = 5 cm; (**B**) chlorophyll content; (**C**) nitrogen content; (**D**) H_2_O_2_ content; (**E**) CAT activity; (**F**) POD activity; (**G**) SOD activity. (n = 3).

Seedlings subjected to 42 °C heat stress revealed that *OsHSP20* is pivotal for thermotolerance (Figure 8). Knockout (*OsHSP20*-KO) lines exhibited severe thermal sensitivity, characterized by a significant decline in chlorophyll and nitrogen content, excessive accumulation of hydrogen peroxide (H_2_O_2_), and a failure to upregulate catalase (CAT) and peroxidase (POD) activities. Conversely, overexpression (*OsHSP20*-OE) lines maintained superior photosynthetic performance and effectively activated CAT and POD activities. Although H_2_O_2_ levels increased in OE lines, the accumulation was attenuated compared to KO lines, correlating with enhanced cellular protection. These results demonstrate that *OsHSP20* mitigates heat-induced oxidative damage and preserves photosynthetic integrity, thereby establishing its critical role in plant thermotolerance.

### 2.9. Transcriptomic Analysis of OsHSP20 Transgenic Lines

**Figure 9:**
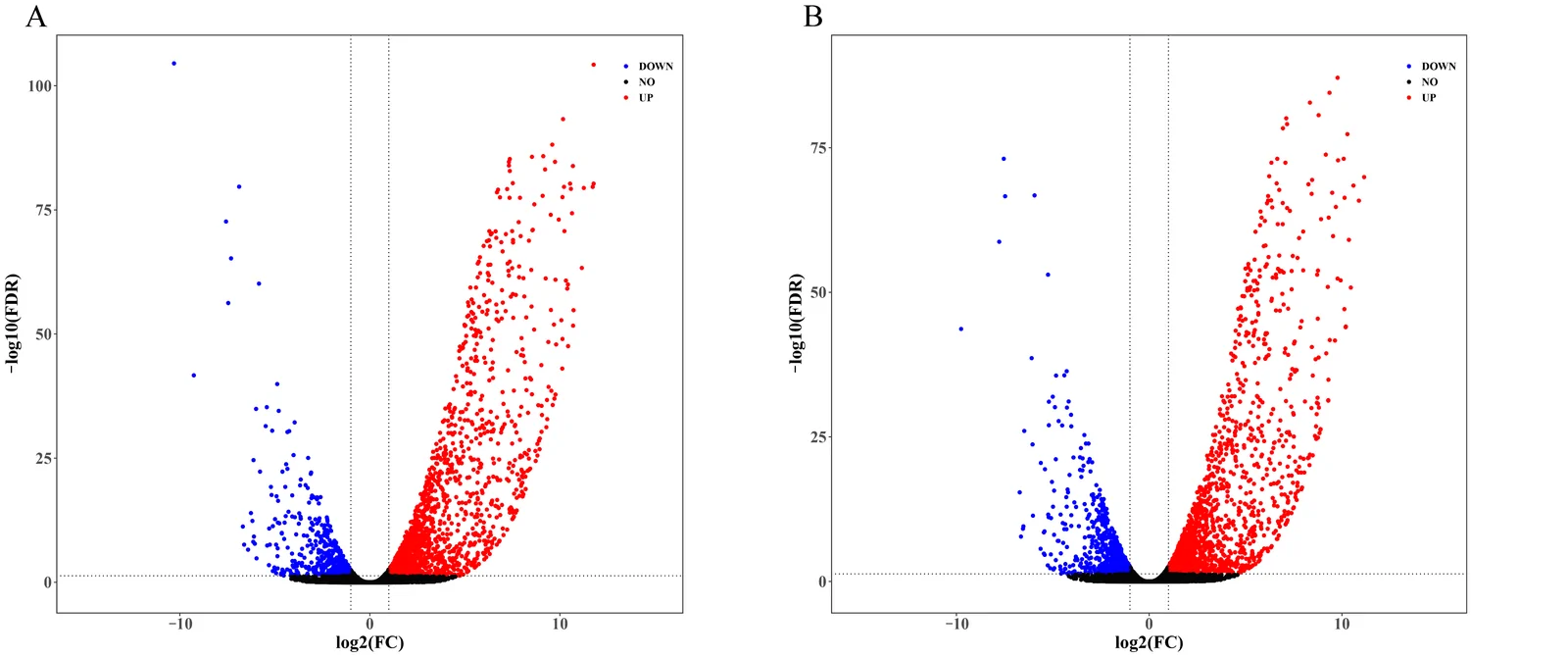
Transcriptomic analysis of *OsHSP20* transgenic lines under soil drought treatment. (**A**) Volcano plot of differentially expressed genes comparing knockout vs. wild-type. (**B**) volcano plot of differentially expressed genes comparing overexpression vs. wild-type.

Transcriptomic analysis of panicle tissues identified 3819 and 3805 differentially expressed genes (DEGs) in *OsHSP20*-KO and *OsHSP20*-OE lines (Figure 9A,B), respectively. GO enrichment indicated that *OsHSP20* primarily governs biological processes, particularly development, reproduction, and stimulus response. Molecularly, DEGs were enriched in protein folding chaperone activity and antioxidant function. KEGG analysis revealed that *OsHSP20* modulates amino sugar/nucleotide sugar metabolism (supporting cell wall synthesis) and starch/sucrose metabolism (determining grain filling) (Figures S1 and S2). Under drought stress, *OsHSP20* reprograms metabolism towards either lipid or cuticular wax biosynthesis, enhancing cuticular barriers to reduce water loss, and activates glutathione metabolism to maintain redox homeostasis (Figures S3 and S4). Concurrently, ABC transporter pathways were regulated, linking carbon allocation to both stress defense and yield formation.

## 3. Discussion

Our results demonstrate that *OsHSP20* is a critical determinant of rice yield, governing panicle architecture, grain filling, and plant stature. The knockout of *OsHSP20* resulted in compromised plant height and increased tillering numbers, whereas overexpression (OE) further intensified tillering quantity and leaf expansion but restored plant height to wild-type (WT) levels [9]. This divergence suggests that *OsHSP20* differentially regulates developmental programs based on gene dosage. These findings align with reports that HSPs function beyond stress tolerance, playing conserved roles in organogenesis and cell cycle progression [10]. The increased tiller number and reduced height in KO lines, contrary to simple loss-of-function expectations, likely reflect functional redundancy within the HSP family or feedback regulation [11,12].

At the reproductive stage, *OsHSP20* deficiency led to severe defects in panicle architecture, including shortened panicles and reduced branch numbers, culminating in a significant decline in grain yield [13]. As molecular chaperones, HSPs ensure the correct folding of key regulatory proteins during the rapid cell division of panicle development [14]. The partial rescue of secondary branches but not primary branches in OE lines suggests a temporal sensitivity in branch meristem determination [15]. Furthermore, the OE-mediated enhancement of grain setting rate, despite a reduction in total grains per panicle, reveals a critical trade-off. This mirrors the increased ineffective tillers observed in OE lines and suggests that *OsHSP20* balances resource allocation between vegetative growth and reproductive output. This trade-off is further evidenced by the reduced 1000-grain weight in OE lines, indicating that excessive chaperone activity may divert resources from storage processes [16].

*OsHSP20* plays a dual role in modulating seedling vigor and drought resilience. KO lines exhibited severe germination defects even under normal conditions, confirming the gene’s essential role in early development [17]. Under drought stress, *OsHSP20* overexpression significantly enhanced root growth compared to WT. However, a nuanced phenotype was observed: while OE lines maintained better shoot growth under stress, they exhibited a more pronounced reduction in plant height compared to WT. This is not a defect but a strategic adaptation. By reducing aerial biomass, the plant minimizes transpirational surface area, thereby conserving water. This morphological adjustment correlates with a higher root-shoot ratio in OE lines, indicative of a prioritized investment in root systems for water acquisition [18].

Physiological analysis revealed that *OsHSP20* fortifies cellular integrity against dehydration [19]. KO lines suffered from severe oxidative damage, characterized by high MDA and H_2_O_2_ accumulation coupled with suppressed antioxidant enzyme (SOD, POD, CAT) activities. In contrast, OE lines maintained robust antioxidant defenses and superior chlorophyll and nitrogen status. This protection extends to thermotolerance, where *OsHSP20* prevented heat-induced chlorosis and ROS bursts by sustaining photosynthetic and antioxidant enzyme complexes. This dosage-dependent protection confirms *OsHSP20* as a central hub in cellular protein quality control [20]. In addition, from our previous study on the discovery of candidate genes for the main effect QTL of photosynthetic rate in rice, we have found that OsHSP20 is one of the four candidate genes for the main effect QTL of photosynthetic rate. The chromosome fragment substitution lines created with it also showed a high photosynthetic rate under drought stress, indicating that this gene has the function of maintaining high photosynthetic efficiency under abiotic stress, thus enabling rice to have high resistance, high photosynthetic efficiency, and high yield under stress such as drought, heat, and light (Supplementary Figure S5).

To determine the subcellular localization of *OsHSP20*, we constructed an *OsHSP20*-GFP green fluorescent fusion vector. The results demonstrated that the fluorescent signal was primarily detected in the nucleus and chloroplasts (Supplementary Figure S6).

Transcriptomic profiling revealed that *OsHSP20* orchestrates a complex network integrating development and stress responses. Under drought, *OsHSP20* reprograms metabolism towards the biosynthesis of cuticular lipids (ether lipids, waxes). This strengthens the epidermal barrier to reduce non-stomatal water loss, explaining the superior water use efficiency in OE lines [21]. Concurrently, *OsHSP20* maintains core metabolic functions, such as starch and sucrose metabolism, ensuring the supply of photoassimilates for grain filling [22].

Furthermore, *OsHSP20* modulates phytohormone signaling and transcriptional regulation. The enrichment of differentially expressed genes in ABA and auxin pathways suggests that *OsHSP20* stabilizes key signaling components, allowing the plant to fine-tune the growth-defense trade-off [23]. Integrating existing knowledge regarding the *OsHSP20* family (Table 1) [7,13,24], this study establishes that *OsHSP20*, functioning as a master regulator orchestrating lipid barrier formation, antioxidant defense, and resource allocation, ultimately dictates the plant’s survival–reproduction strategy under adverse conditions.

## 4. Materials and Methods

### 4.1. Construction and Identification of Transgenic Rice Lines

To generate *OsHSP20* knockout (*OsHSP20*-KO) lines using CRISPR/Cas9 genome editing, an sgRNA targeting the exon sequence of *OsHSP20* (*LOC_Os10g30162.1*) was designed and inserted into the pHSbdcas9i plasmid to construct the knockout vector. The vector was introduced into rice via genetic transformation, and knockout lines were obtained through screening and identification. For overexpression, the coding sequence (CDS) of *OsHSP20* was cloned into the pBWA(V)HS overexpression vector driven by the CaMV 35S promoter, and overexpression lines were generated through rice genetic transformation, screening, and identification. The identification results of positive bacterial clones and positive rice lines are shown in Figure S7.

### 4.2. Plant Growth Conditions and Agronomic Trait Investigation

The japonica rice cultivar Zhonghua 11 (ZH11) and its derived *OsHSP20* knockout (KO) and overexpression (OE) lines were used in this study. Plants were cultivated in pots under natural field conditions at the Experimental Farm of the College of Bioscience and Bioengineering, Jiangxi Agricultural University, China. At the mature stage, ten plants per line were randomly selected to investigate agronomic traits, including plant height, tiller number, panicle architecture, and grain-related characteristics. All data were collected from ten biological replicates per genotype.

### 4.3. Drought and Heat Stress Treatments

Seed Germination Assay: Dehusked and sterilized seeds were germinated on Murashige and Skoog (MS) medium supplemented with or without 150 mM mannitol. Plates were incubated at 28 °C under a 14 h/10 h light/dark cycle.

Post-Germinative Drought Stress: Seedlings grown on 1/2 MS medium were transferred to hydroponic boxes containing 1/4 strength rice nutrient solution with or without 150 mM mannitol. Shoot and root lengths were measured daily.

Vegetative Stage Drought Stress: Four-leaf-stage seedlings were treated with 20% polyethylene glycol (PEG) 6000 in hydroponic solution for 5 days under controlled conditions (30 °C, 75% humidity).

Soil Drought Stress: Potted tillering-stage plants were subjected to water withholding for 12 days. Leaf samples were collected for physiological analysis, and soil water content was determined. Three biological replicates were included per group.

High-Temperature Stress: Four-leaf-stage seedlings were exposed to 42 °C (treatment) or 30 °C (control) for 3 days. The third fully expanded leaf was harvested for biochemical measurements.

### 4.4. Physiological and Biochemical Measurements

Chlorophyll and nitrogen contents in rice leaves were measured using a chlorophyll meter (Model YT-YD, Shandong Yuntang Intelligent Technology Co., Ltd., Weifang, China). For oxidative stress assessments, fresh leaf samples were collected, and the contents of hydrogen peroxide (H_2_O_2_) and malondialdehyde (MDA), as well as the activities of peroxidase (POD), catalase (CAT), and superoxide dismutase (SOD), were quantified using commercial assay kits (Suzhou Comin Biotechnology Co., Ltd., Suzhou, China) according to the manufacturer’s instructions. All measurements were performed with three biological replicates.

### 4.5. Transcriptome Sequencing and Bioinformatics Analysis

Young panicle tissues were collected and sent to Shanghai Lingen Biotech Co., Ltd. (Shanghai, China) for Illumina paired-end RNA sequencing. Raw sequencing data were processed and analyzed using the online cloud platform provided by the company (http://cloud.biomicroclass.com/CloudPlatform, URL accessed on 29 November 2025). Differential expression analysis and functional enrichment were performed according to the platform’s standard pipeline.

### 4.6. Statistical Analysis

All experimental data were analyzed using GraphPad Prism 10.1.2 software. Values are expressed as the mean ± standard deviation (SD) of three biological replicates. Statistical significance was evaluated using one-way or two-way analysis of variance (ANOVA), followed by Tukey’s honest significant difference test. Significant differences among groups are indicated by different lowercase letters above the bars (*p* < 0.05).

## 5. Conclusions

This study establishes *OsHSP20* as a pivotal regulator coordinating rice growth, yield, and stress resilience. Using gene knockout and overexpression lines, we demonstrate that *OsHSP20* exerts pleiotropic effects: its deficiency causes dwarfism, excessive tillering, and severe yield loss due to impaired panicle architecture, while its overexpression enhances grain setting rate. Crucially, *OsHSP20* acts as a positive modulator of drought and heat tolerance. Loss of function leads to catastrophic oxidative damage and failed antioxidant activation, whereas overexpression fortifies cellular defense systems. Mechanistically, transcriptomic analysis reveals that *OsHSP20* functions as a core molecular chaperone. It safeguards protein homeostasis to coordinate a network integrating lipid barrier formation, antioxidant defense, and carbon allocation. We propose that *OsHSP20* optimizes crop fitness by balancing resource investment between growth and stress adaptation. This work identifies *OsHSP20* as a valuable genetic resource for rice molecular breeding aimed at high yield, stress resilience and high photosynthetic rate. For example, the more than 20 chromosome fragment substitution lines obtained by backcrossing with Zhenshan 97B using molecular marker-assisted selection are typical high-yield, high-resistance, and high-photosynthetic-efficiency rice germplasm resources of this type.

## Figures and Tables

**Table 1:** Functional characteristics of *OsHSP20* family members.

Gene ID	Chromosome	Function and Subcellular Localization
*Os01g0135800*	Chr 1	Cytosolic localization; indirectly alleviates ER folding burden by restricting cytosolic protein aggregation, thereby maintaining protein homeostasis.
*Os01g0136200*	Chr 1	Cytosolic localization; responds to heat stress.
*Os02g0782500*	Chr 2	Cytosolic/nuclear localization; tightly co-clusters with *OsHsp17.7*/*OsHsp17.3*.
*Os03g0245800*	Chr 3	Cytosolic localization; responds to heat stress.
*Os03g0266900*	Chr 3	Cytosolic localization; responds to heat stress.
*Os03g0267000*	Chr 3	Cytosolic localization; molecular chaperone; inhibits heat-induced MDH aggregation, enhances thermotolerance; improves salt tolerance; non-responsive to drought/ABA.
*Os03g0267200*	Chr 3	Cytosolic localization; responds to heat stress.
*Os04g0445100*	Chr 4	Involved in protein stabilization and maintenance of proper folding under high temperatures, preventing aggregation of heat-denatured proteins.
*Os11g0244200*	Chr 11	Possesses protein chaperone activity; strongly upregulated under high temperatures to maintain protein stability and prevent aggregation.
*Os12g0514500*	Chr 12	Co-clusters with OsBiP3.

## Data Availability

The original contributions presented in this study are included in the article/Supplementary Materials. Further inquiries can be directed to the corresponding authors.

## Supplementary Materials

The following supporting information can be downloaded at: https://www.mdpi.com/article/10.3390/ijms27188341/s1.

## Abbreviations

HSP (Heat Shock Protein), KO (Knockout), OE (Overexpression), WT (Wild Type), DEG (Differentially Expressed Gene), GO (Gene Ontology), KEGG (Kyoto Encyclopedia of Genes and Genomes), ROS (Reactive Oxygen Species), SOD (Superoxide Dismutase), POD (Peroxidase), CAT (Catalase), MDA (Malondialdehyde), PEG (Polyethylene Glycol), MS (Murashige and Skoog medium), qPCR (Quantitative PCR), SRA (Sequence Read Archive), GEO (Gene Expression Omnibus), GSA (Genome Sequence Archive), CK (Control), DS (Drought Stress).
