# Supplementary material for: Dual Regulation of the Rice Yield Traits and Stress Resilience by OsHSP20: Insights from Integrative Biochemical and Transcriptomic Analyses
Source: Int J Mol Sci. 2026 Sep 19;27(18):8341. doi: 10.3390/ijms27188341 (PMC13607055; doi:10.3390/ijms27188341)
Supplement: Supplementary file 1 [file ijms-27-08341-s001.zip › Supplementary Materials-Correct version.pdf]

Supplementary Materials:

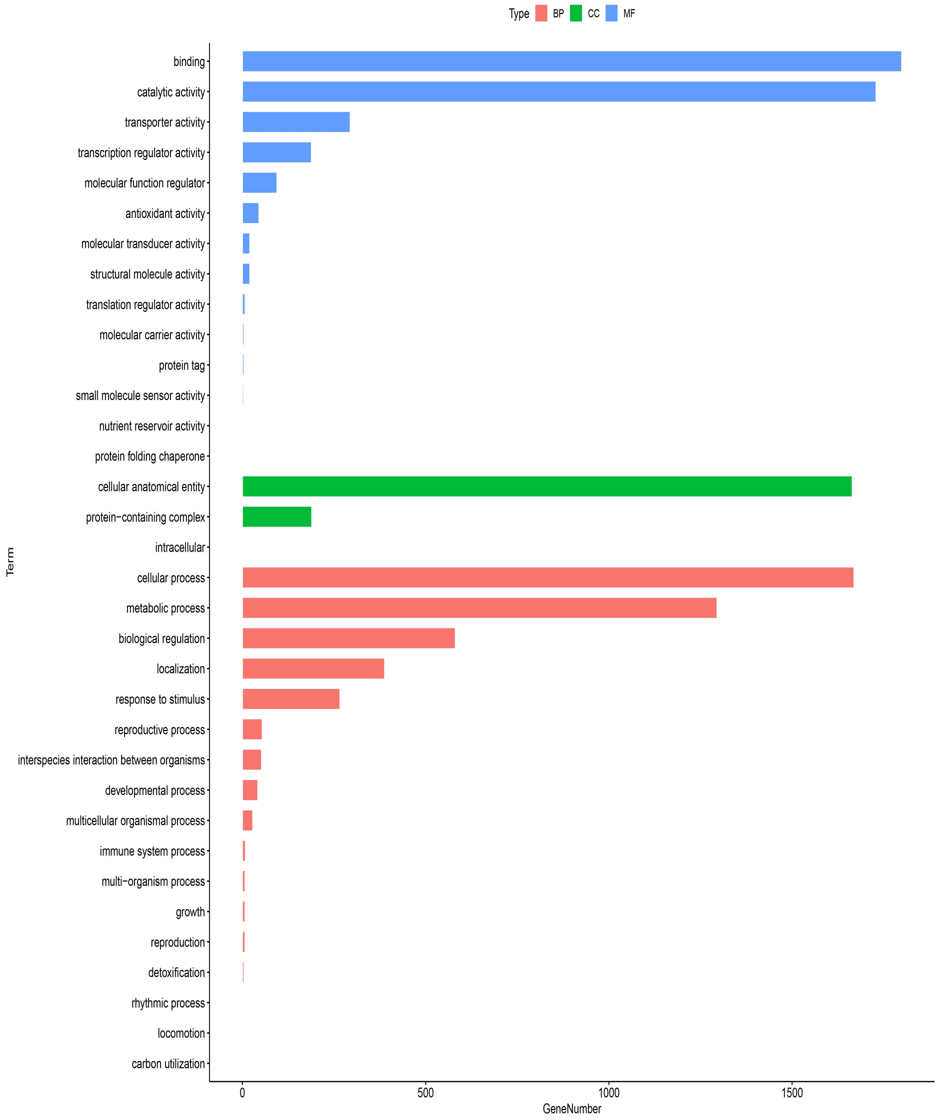

Supplementary Figure S1. Gene Ontology (GO) enrichment bar plot for DEGs between transgenic lines under normal conditions.

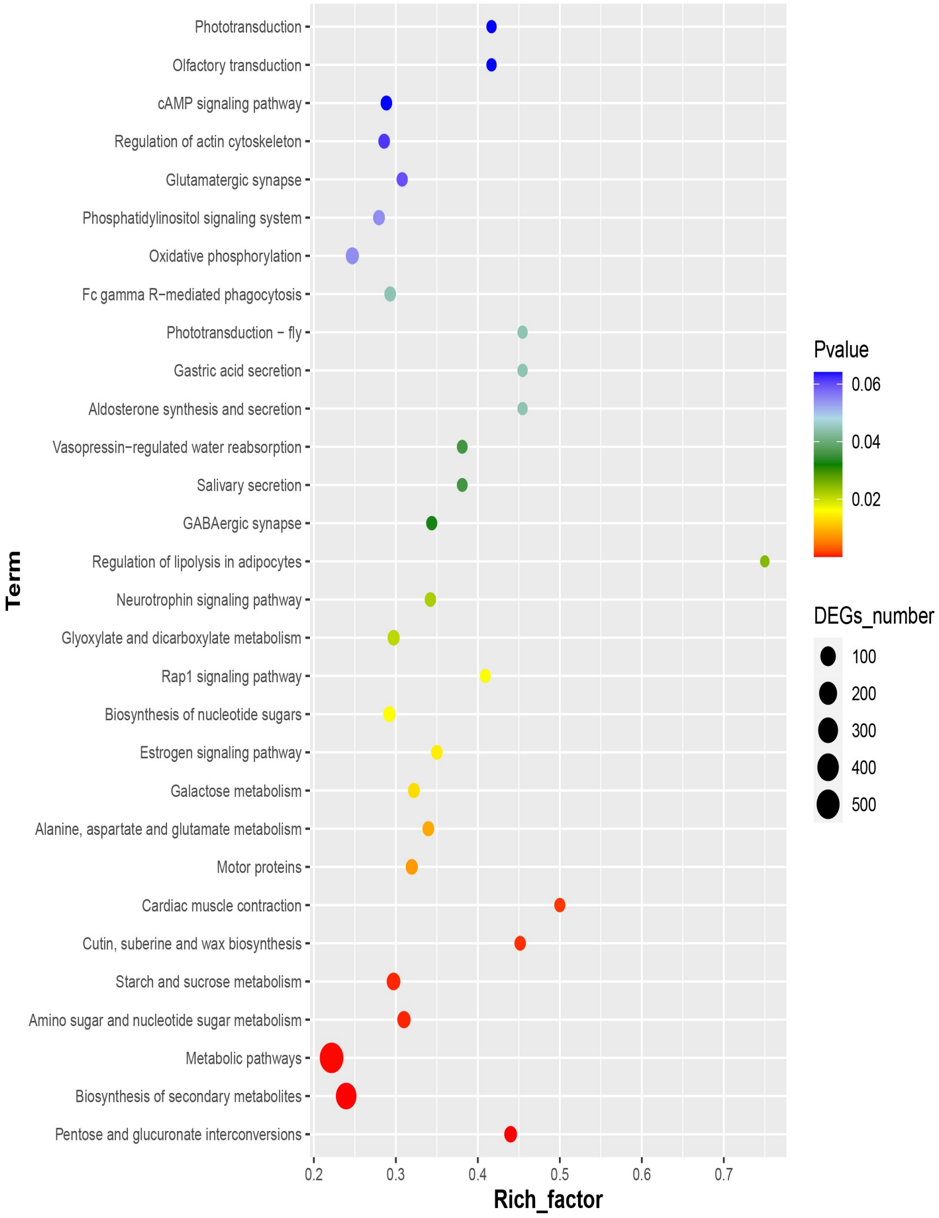

Supplementary Figure S2. Kyoto Encyclopedia of Genes and Genomes (KEGG) pathway enrichment bubble chart for DEGs between transgenic lines under normal conditions.

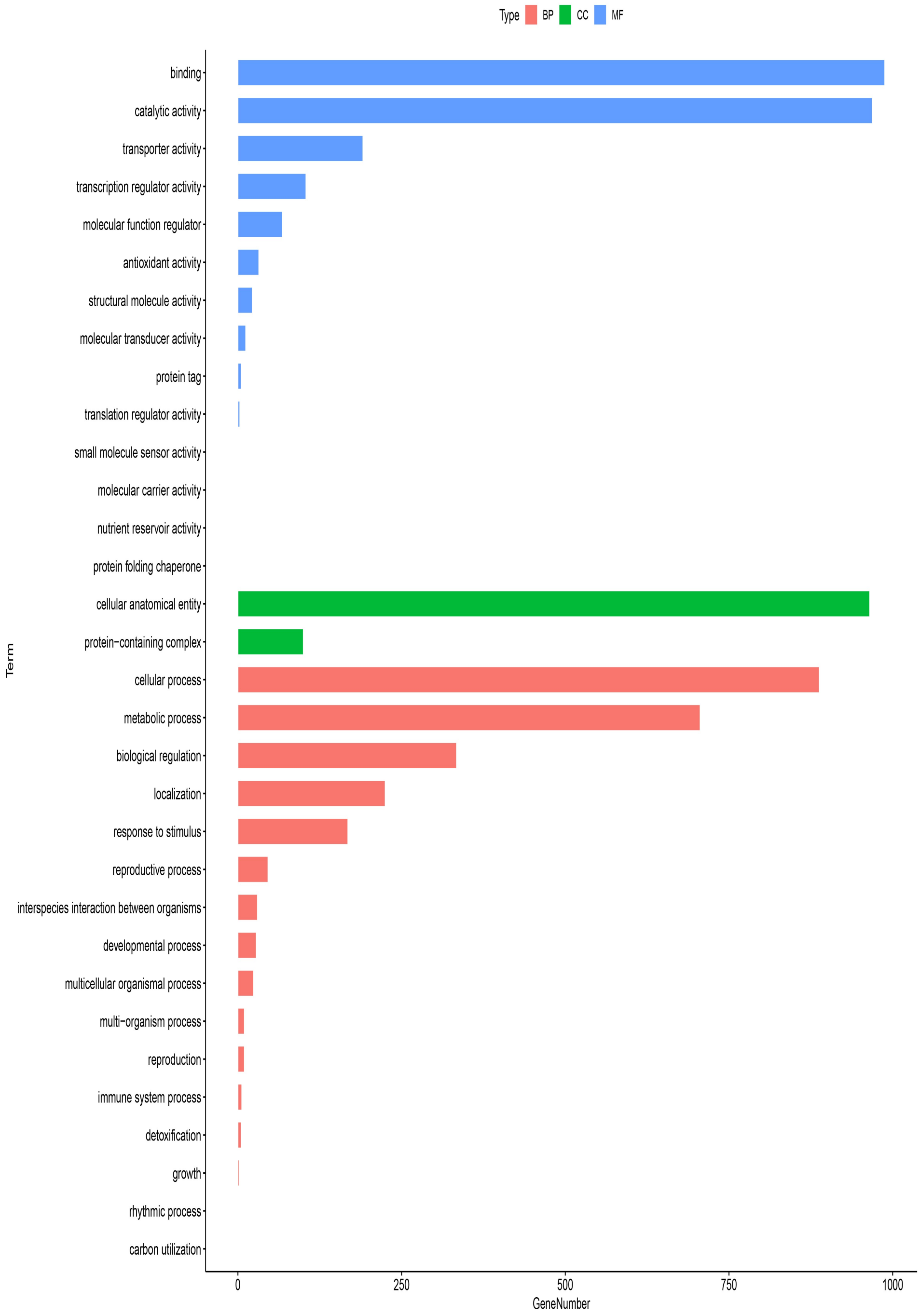

Supplementary Figure S3. GO enrichment bar plot for DEGs between transgenic lines under drought stress.

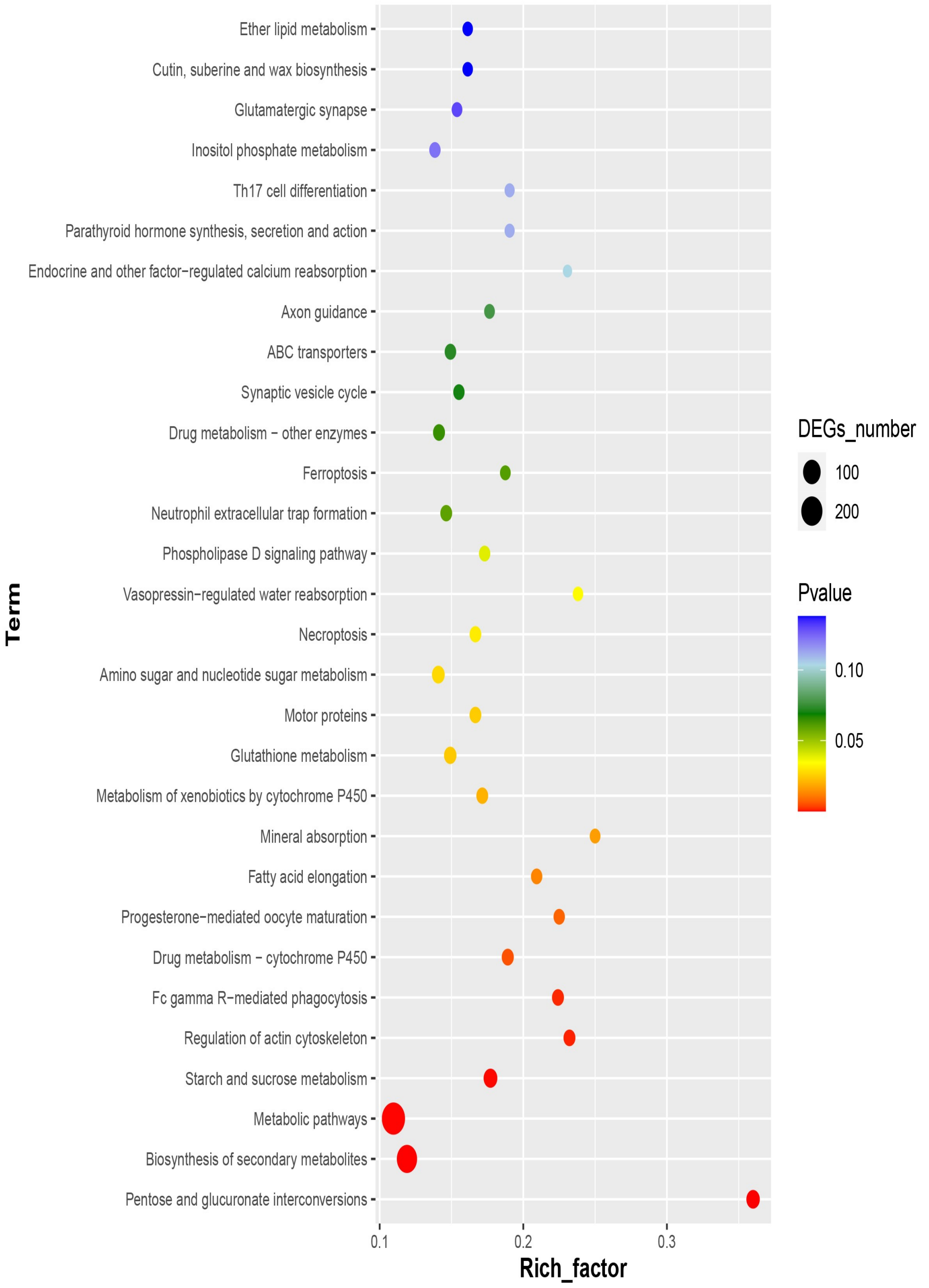

Supplementary Figure S4.KEGG pathway enrichment bubble chart for DEGs between transgenic lines under drought stress.

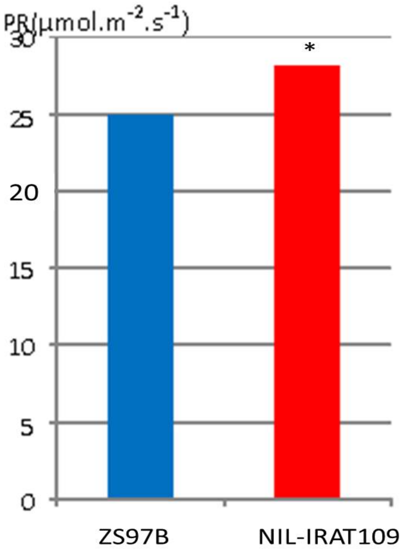

Supplementary Figure S5. Significant difference analysis of PR between ZS97B and NIL-IRAT109.

Notes: PR means photosynthetic rate. This figure shows the results of photosynthetic rate measurements of 9 individual plants carrying IRAT109 donor fragments and 9 maternal plants of Zhenshan 97B selected from the BC3F2 population after fully unfolding the sword leaves during the booting stage, with significant differences at the level of significance(0.0286) and the statistical test was used for this comparison (n=9 each group).

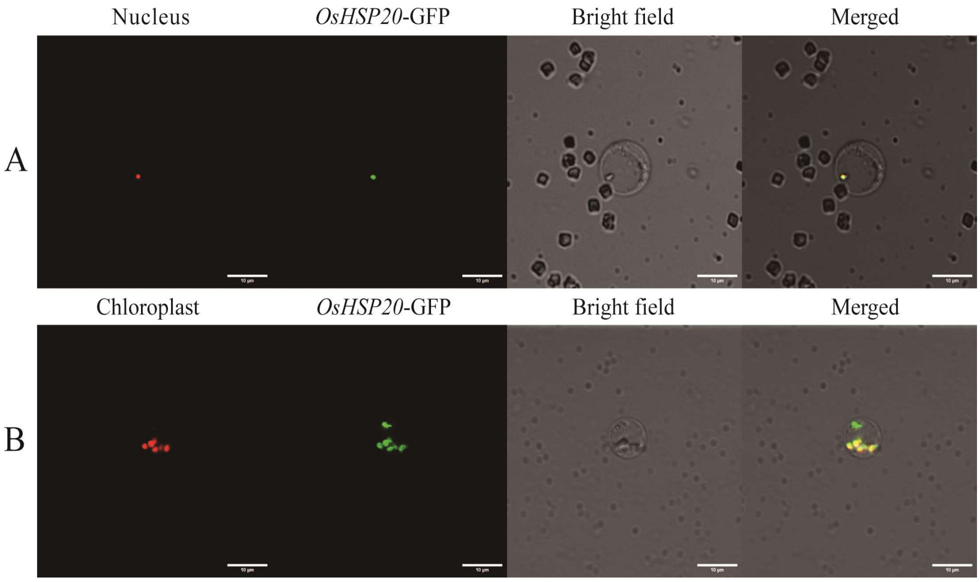

Supplementary Figure S6. Subcellular localization of *OsHSP20*. (A) Fluorescent signal indicating nuclear localization；(B) fluorescent signal indicating chloroplast localization.Scale bar=10μm.

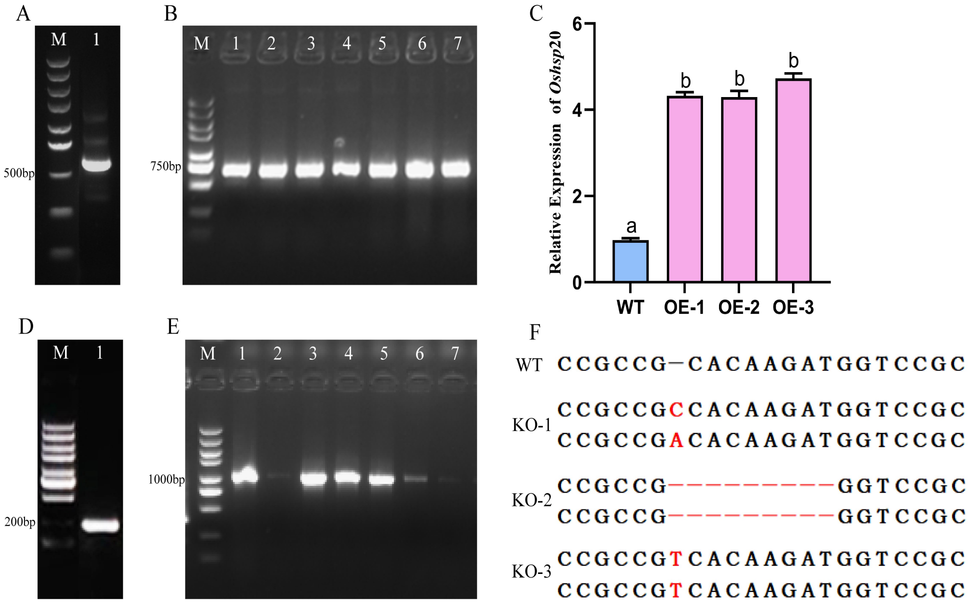

Supplementary Figure S7. Construction and identification of *OsHSP20* transgenic lines. (A) Target fragment of the overexpression vector; (B) PCR validation of the overexpression vector in bacterial culture; (C) transcript level validation of the overexpression lines; (D) target fragment of the knockout vector; (E) PCR validation of the knockout vector in bacterial culture; (F) sequencing results of the knockout lines. (n=3).
